# *DMD* genotype correlations from the Duchenne Registry: Endogenous exon skipping is a factor in prolonged ambulation for individuals with a defined mutation subtype

**DOI:** 10.1002/humu.23561

**Published:** 2018-07-12

**Authors:** Richard T. Wang, Florian Barthelemy, Ann S. Martin, Emilie D. Douine, Ascia Eskin, Ann Lucas, Jenifer Lavigne, Holly Peay, Negar Khanlou, Lee Sweeney, Rita M. Cantor, M. Carrie Miceli, Stanley F. Nelson

**Affiliations:** ^1^ Department of Human Genetics David Geffen School of Medicine University of California ,Los Angeles California; ^2^ Center for Duchenne Muscular Dystrophy University of California, Los Angeles,Los Angeles California; ^3^ Department of Microbiology, Immunology, and Molecular Genetics, David Geffen School of Medicine and College of Letters and Sciences University of California, Los Angeles, Los Angeles California; ^4^ Parent Project Muscular Dystrophy Hackensack New Jersey; ^5^ RTI International Research Triangle Park North Carolina; ^6^ Department of Pathology and Laboratory Medicine, David Geffen School of Medicine University of California Los Angeles California; ^7^ Department of Pharmacology and Therapeutics University of Florida Gainesville Florida; ^8^ Molecular Biology Institute University of California, Los Angeles California Los Angeles

**Keywords:** Duchenne muscular dystrophy, Duchenne Registry, rare disease registry

## Abstract

Antisense oligonucleotide (AON)‐mediated exon skipping is an emerging therapeutic for individuals with Duchenne muscular dystrophy (DMD). Skipping of exons adjacent to common exon deletions in *DMD* using AONs can produce in‐frame transcripts and functional protein. Targeted skipping of *DMD* exons 8, 44, 45, 50, 51, 52, 53, and 55 is predicted to benefit 47% of affected individuals. We observed a correlation between mutation subgroups and age at loss of ambulation in the Duchenne Registry, a large database of phenotypic and genetic data for DMD (*N* = 765). Males amenable to exon 44 (*N* = 74) and exon 8 skipping (*N* = 18) showed prolonged ambulation compared to other exon skip groups and nonsense mutations (*P* = 0.035 and *P* < 0.01, respectively). In particular, exon 45 deletions were associated with prolonged age at loss of ambulation relative to the rest of the exon 44 skip amenable cohort and other *DMD* mutations. Exon 3–7 deletions also showed prolonged ambulation relative to all other exon 8 skippable mutations. Cultured myotubes from DMD patients with deletions of exons 3–7 or exon 45 showed higher endogenous skipping than other mutations, providing a potential biological rationale for our observations. These results highlight the utility of aggregating phenotypic and genotypic data for rare pediatric diseases to reveal progression differences, identify potentially confounding factors, and probe molecular mechanisms that may affect disease severity.

## 1 INTRODUCTION

Duchenne muscular dystrophy (DMD, MIM # 310200) is a fatal X‐linked disease characterized by a progressive loss of skeletal muscle function. It is most commonly caused by large deletions in *DMD* resulting in loss of dystrophin expression. With a prevalence of 1 in 3,500–5,000 live male births (Center for Disease Control and Prevention (CDC), 2009; Mathews et al., 2010), it is the most common pediatric muscular dystrophy. Single or multiple exonic deletions and duplications account for 80% of mutations that cause DMD and the allelic disorder Becker muscular dystrophy (BMD; Bladen et al., 2015). Age at loss of ambulation (LOA) is variable in DMD, but typically, steroid‐naïve boys lose independent walking ability between 9 and 11 years while those treated with corticosteroids ambulate on average for an additional 2–3 years (Angelini et al., 1994; Griggs et al., 1991, 1993; Henricson et al., 2013; Mendell et al., 1989; Wang et al., 2014). Steroid benefit has been clearly demonstrated in clinical trials, meta‐analysis, and multiple natural history studies, and it has been the main drug intervention in DMD to date (Mendell et al., 1989).

Recently, AON‐mediated exon‐skipping strategies for DMD have shown promise in restoring myofiber expression of dystrophin and slowing disease progression (Goemans et al., 2011; van Deutekom et al., 2007). The dystrophin protein encoded by the 79 exons of the *DMD* transcript is among the largest in the proteome and consists of an N‐terminal actin binding domain, 24 spectrin‐like repeats, a cysteine‐rich domain, and a C‐terminal domain (Aartsma‐Rus, Van Deutekom, Fokkema, Van Ommen, & Den Dunnen, 2006). Like DMD, the milder BMD is also often caused by large deletions within *DMD* but usually results in a preserved reading frame that results in expression of some functional protein (Monaco, Bertelson, Liechti‐Gallati, Moser, & Kunkel, 1988). AON‐induced exon skipping restores the reading frame in DMD by targeting specific exons for exclusion by the splicing machinery (Goemans et al., 2011; Koenig et al., 1989; van Deutekom et al., 2007). This has been shown to produce correctly localized functional dystrophin in mice (Mann et al., 2001), dogs (Yokota et al., 2009), and humans (Cirak et al., 2011). Targeting of exons 8, 44, 45, 50, 51, 52, 53, and 55 is predicted to help roughly 4%, 8%, 13%, 5%, 15%, 3%, 9%, or 2% of affected DMD boys, respectively (van Deutekom et al., 2001). However, the effect size for each of these AONs is not clear.

Aside from the “reading frame hypothesis,” which demonstrated out‐of‐frame *DMD* exonic deletions typically caused DMD while in‐frame deletions produced the milder BMD (Aartsma‐Rus et al., 2006; Koenig et al., 1989), no concrete rules correlating frameshifting deletions in the mutational hotspot region and disease severity have emerged. However, exceptions to the reading frame rule exist: for instance, out‐of‐frame deletions of exons 3–7 sometimes result in a BMD phenotype, while in‐frame deletions of exon 3 can exist in patients with a DMD phenotype (Kesari et al., 2008; Koenig et al., 1989; Tuffery‐Giraud et al., 2009). Recently, some DMD individuals with mutations amenable to exon 44 skipping were observed to have a higher rate of revertant fibers and a larger number of trace dystrophin positive fibers relative to muscles from exon 51 amenable DMD boys (Lourbakos et al., 2011). Consistent with this description, several studies have indicated that exon 44 skip amenable DMD boys have an overall better functional outcome with higher age at LOA relative to typical DMD (Bello et al., 2016; Pane et al., 2014; van den Bergen, Ginjaar, Niks, Aartsma‐Rus, & Verschuuren, 2014).

We sought evidence of correlation between age at LOA and genetic mutation subgroups in a large cohort of predominantly U.S. DMD patients using data available from the Duchenne Registry, a large patient‐powered self‐report registry for individuals or families affected by Duchenne and BMD (Rangel, Martin, & Peay, 2012; Wang et al., 2014). We data mined patient‐reported parameters of age, ambulatory status, age at LOA, corticosteroid usage, and genetic mutation from 3,383 participants in the registry and corrected for the potential confounding effects of steroid usage. Patients with mutations correctable by skipping exon 8 or 44 ambulated significantly longer than deletion mutations correctable by skipping of exons 45, 50, 51, 52, 53, or 55, exon duplications, or nonsense deletions. The observed delay of age at LOA in boys with exon 44 targetable mutations was primarily due to patients with exon 45 deletion. Interestingly, boys with mutations correctable by skipping exon 51 lost ambulation earlier than the rest of the study cohort, and specific mutation types had different ages at LOA.

These clinical measures are supported by molecular evidence of endogenous exon skipping from cultured myotubes derived from patients at the Center for Duchenne Muscular Dystrophy (CDMD) at the University of California, Los Angeles. Significantly higher basal exon skipping was observed in myotubes derived from individuals with deletions of exon 45 or exons 3–7 compared to those with deletions of exons 45–50 or 49–50, indicating a potential explanatory cause for the biostatistical results.

## 2 MATERIALS AND METHODS

### 2.1 Data collection

**Figure 1:**
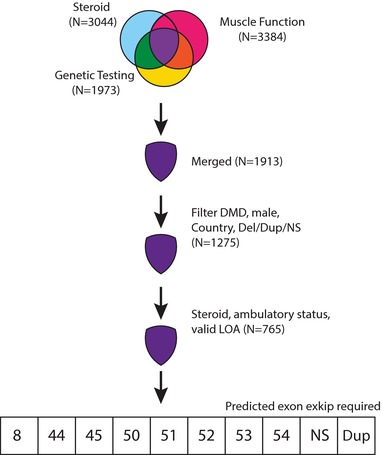
Filtering steps of Duchenne Registry data for analysis in this study. Participants can respond to one or more survey modules on steroid use, genetic testing results, or muscle function. Data for individuals who responded to all three submodules were merged. We removed entries that did not include valid diagnosis, mutation type, country of residence, steroid usage, ambulatory status, and age at LOA status. Individuals amenable to exon skipping were then sorted into the predicted exon skip required to generate in‐frame *DMD* transcript

The Duchenne Registry is an online self‐report registry (http://www.duchenneregistry.org) for individuals and families affected by Duchenne and BMD (Rangel et al., 2012). Participants respond to questionnaires for specific topics: diagnosis, muscle function, corticosteroid, cardiac, respiratory, family history, and genetic testing. Genetic testing reports are reviewed by certified genetic counselors and annotated in a standard format. We downloaded the deidentified October 2016 freeze of the Duchenne Registry dataset which contains responses from 108 countries. Preliminary quality control and filtering were performed as previously described (Wang et al., 2014). Briefly, we filtered the data for males who reported valid and consistent ambulation status, corticosteroid usage/nonusage, and genetic mutation results confirmed by submission of genetic report to the Duchenne Registry genetic counselors. Participants were limited to residents of member nations of the Organization for Economic Cooperation and Development to ensure comparable levels of health care quality and access. For this analysis, genetic testing, corticosteroid, and muscle function modules were assembled per individual resulting in 1,913 complete profiles (Figure 1). Patients were then stratified into separate groups according to either the predicted exon skip necessary to produce an in‐frame transcript (exon 8, 44, 45, 50, 51, 52, 53, or 55), possession of frameshifting exonic duplication or nonsense mutation or other exonic deletions not currently targetable by exon skipping.

All participants of the Duchenne Registry consented during registration for their deidentified data to be shared with researchers. Downloaded, deidentified data were deemed exempt by University of California Los Angeles Institutional Review Board.

### 2.2 Statistical analysis

Kaplan–Meier analysis was used to assess differences in age at LOA among the Duchenne Registry respondents when grouped by varying genetic mutations. Individuals considered in our analysis were currently using corticosteroids, possessed a defined mutation and a valid age at LOA. Age at LOA was defined as the age at which a boy required full‐time wheelchair use as entered in the Duchenne Registry database. Ambulatory participants were included as right‐censored data to augment statistical power. Age at LOA has been used by several other studies because it is a significant milestone and is well recalled by patients or their families. Log‐rank test was used to statistically test for differences between Kaplan–Meier plots of age at LOA among genetic mutation groupings. Cox proportional hazards regression was used to estimate the effect of individual variables on age at LOA. Differences due to mutation subgroups were quantified as hazard ratios (HR), with confidence intervals (CI). HR < 1 indicated delayed age at LOA. We included the same mutational subgroups above regardless of corticosteroid status while using age at LOA as outcome variable. Statistical analysis was performed using R version 3.3.1 (64 bit) and version 2.37 of the Survival package. Kaplan–Meier analysis on steroid naïve patients was not attempted due small sample sizes.

### 2.3 Collection, isolation, and propagation of dermal fibroblasts and myoblasts

Skin punches were obtained with informed consent from patients of the CDMD under University of California Los Angeles IRB‐approved protocol (#11‐001087). Isolation of dermal fibroblasts followed published protocols (Karumbayaram et al., 2012). Cells were reprogrammed using a tamoxifen‐inducible MyoD overexpression system as previously described (Kendall et al., 2012) to create induced directly reprogrammed myotubes (iDRM). Primary myoblasts were purified from 50–100 mg of tissue obtained by needle muscle biopsy of vastus lateralis. Each core biopsy was dissociated into 1‐mm pieces in a 1:1 MIX of dispase (1.5 U/mL)/collagenase (1,000 U/mL). After 20 minutes at 37°C, chunks were triturated and passed through a 70‐μm cell strainer. Cell suspension was centrifuged at 1,200 rpm for 4 minutes, and the pellet was resuspended in 10 mL growth media (Nutrient Mixture F‐10 HAM with 20% FBS and 1% pen/strep) and plated into a T75 flask. After 1 hour of preplating, the cell suspension was placed into a new T75 flask, which was considered to be enriched in myoblasts. All derived cells are assigned a patient unique study ID to allow inclusion of clinical data including mutation type and clinical progression.

### 2.4 In‐culture myotube formation

Inducible directly reprogrammable myotubes (iDRMs) were seeded at 200,000 cells per well in fibroblast growth media (DMEM (+phenol red, high glucose) + 15% FBS + 1% nonessential amino acids + 1% pen/strep) in 6‐well plates (Corning) precoated for 1 hour with 0.1% gelatin (Sigma). The following day, 5 μM 4OH‐tamoxifen (Sigma; resuspended in ethanol) was added in fibroblast growth media for 24 hours. On day 3, cells were washed in 1× phosphate‐buffered saline (PBS; Invitrogen), and fusion media containing 1 μM 4OH‐tamoxifen was added (1:1 Ham's F‐10:DMEM (phenol red free, high glucose), 2% horse serum, 2% insulin–transferrin–selenium). On day 7, cell pellets were harvested and frozen for subsequent RNA isolation and endogenous exon‐skipping analysis via nested PCR with primers encompassing the deleted region.

Primary myoblasts were cultivated in growth media as described above but supplemented with 5 ng/mL of bFGF starting at day 3. The media were changed every 3 days until confluence was reached. At confluence, media were exchanged for skeletal muscle differentiation media (Promocell) for 7 days before being harvested in TRIzol for RNA isolation (Ambion).

### 2.5 RNA isolation, PCR, and quantification

Total RNA was isolated using the Purelink RNA mini kit (Ambion). For exon‐skipping analysis, 200–500 ng of total RNA was reverse transcribed with random hexamers (Life Technologies). For cell lines with a deletion of exon 45, nested PCR was performed between exons 42–46 (Ex42‐o: CAATGCTCCTGACCTCTGTGC + Ex 46‐o: GCTCTTTTCCAGGTTCAAGTGG and Ex 43‐i: GTCTACAACAAAGCTCAGGTCG + Ex 46‐i: GCAATGTTATCTGCTTCCTCCAACC). For cell lines with a deletion of exons 45–50, we used primers previously described (Kendall et al., 2012). For cell lines with a deletion of exons 49–50 or 48–50, a nested PCR was performed between exons 42 and 46 (Ex47‐o: AGGACCCGTGCTTGTAAGTG + Ex 55‐o: TCTTCCAAAGCAGCCTCTCG and Ex 47‐i: AGCAGACAAATCTCCAGTGGA + Ex 53‐i: TTCAACTGTTGCCTCCGGTT). After the verification on agarose gel, samples are loaded on a DNA1000 Bioanalyzer chip and run on the 2100 Bioanalyzer (Agilent Technologies). This technology plots fluorescence intensity versus migration time and produces an electropherogram for each sample. Results are analyzed by quantitating the molarity of products within the peaks at the expected sizes. Percentage of exon skipping was calculated as the molar amounts of  skipped  PCR  product  skipped  PCR  product + unskipped  PCR  product ×100. For cell lines with a deletion of exons 3–7, primers were used as previously described (Fletcher et al., 2012).

### 2.6 Immunofluorescence on cultured myotubes

Primary myoblasts were grown as described above. After induction of differentiation for 7 days, cells were fixed with acetone for 7 minutes at −20°C. Dystrophin was detected using MANDYS8 (directed against the central rod domain, sc‐58754, Santa Cruz Biotechnology) at a dilution of 1:100. Secondary goat anti‐mouse (A32731, Life Technologies) was used at a dilution of 1:500. Images were obtained using a Zeiss microscope at a 20× magnification and processed with Axiovision software and/or ImageJ software.

### 2.7 Muscle biopsy immunohistochemistry

Muscle biopsies were flash frozen in liquid nitrogen–cooled isopentane and stored at −80°C. At the time of processing, biopsies were mounted in OCT (Tissue‐Tek) and 10‐μm transverse cryosections were obtained. These were treated in 3% aqueous solution of hydrogen peroxide for 10 minutes and were blocked with 2.5% normal horse serum (Vector S2012) for 30 min at room temperature. Sections were incubated in primary antibody at 37°C degrees for 1.5 hours (dystrophin rod domain: NCL‐DYS1, 1:50, Leica Biosystems; dystrophin C‐terminal NCL‐DYS2, 1:50, Leica Biosystems) and (dystrophin 3: NCL‐DYS3, 1:50, Leica Biosystems; antibody diluent: DAKO S3022). Subsequently, the slides were rinsed in PBS and placed in secondary antibody for 1 hour. The sections were developed using DAB reagent (DAB substrate ki9t, vector SK‐400), counterstained in hematoxylin for 2 minutes, dehydrated in graded alcohol and xylene, and coverslipped with Permount. Images were obtained as for cultured myotubes.

## 3 RESULTS

Kaplan–Meier analysis was performed on males with DMD who were currently using corticosteroids and had deletion mutations amenable to targeted skipping of exons 8, 44, 45, 50, 51, 52, 53, or 55, as well as exonic duplications or nonsense mutations. Age at LOA, defined as the age at which a male with DMD required full‐time wheelchair use, was the outcome variable.

**Figure 2:**
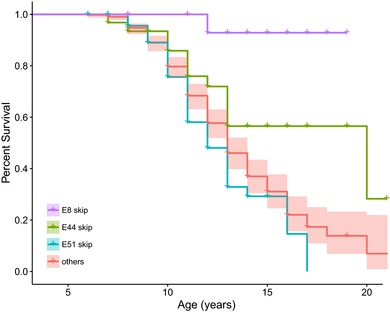
Kaplan–Meier age at LOA analysis for patients eligible for skipping therapy of exons. Delayed age at LOA was observed among individuals amenable to exon 8 skipping (*P* < 0.001) and exon 44 skipping (*P* = 0.04). Exon 51 skippable individuals had earlier age at LOA (*P* = 0.04). All other groups (45, 50, 52, 53, duplication and nonsense) were not significantly different and were merged. All subjects were currently using corticosteroids

Three groups showed statistically significant differences in age at LOA compared to the remainder of the group by a log‐rank test (Figure 2). Individuals potentially amenable to exon 44 skipping therapy (e44 skip; *N* = 74) showed a difference in age at LOA by the log‐rank test (*P* = 0.035), with a striking median age at LOA of 20 years, in contrast to the median age at LOA of 13 for the remainder of the cohort. The large majority of boys amenable to exon 8 targeted therapy (*N* = 18) were still ambulatory at age 20 (*P* < 10^−5^), and most of these boys have exon 3–7 deletion, which is often affiliated with a BMD phenotype (Muntoni et al., 1994). Consistent with previously reported cohort studies of DMD, the largest group consisted of individuals amenable to exon 51 therapy (e51 skip; *N* = 106). This group has a more severe disease progression with an observed earlier median age at LOA at age 12 (*P* = 0.04). No other DMD mutation subgroups showed significant differences in age at LOA (Table 1, Supporting Information Figure S1).

**Table 1:** Table of mutation subgroups and log‐rank test *P* values

Mutation subgroup	*N*	%	Median age at LOA (years)	Log‐rank *P*
Exon 8 skippable	18	2.4	NA	**<0.01**
Exon 44 skippable	74	9.7	20	**0.04**
Exon 45 skippable	70	9.1	13	0.80
Exon 50 skippable	33	4.3	16	0.24
Exon 51 skippable	106	13.8	12	**0.04**
Exon 52 skippable	29	3.8	16	0.52
Exon 53 skippable	78	10.2	12	0.62
Exon 55 skippable	21	2.7	13	0.24
Duplication	83	10.8	13	0.50
Nonsense	71	9.3	14	0.59
All other exonic deletions	182	23.8	13	NA

Log‐rank *P* value is for comparison between specific subgroup compared to all other subgroups in aggregate. Significant tests with *P* < 0.05 are bolded. *N* denotes number of individuals. LOA, loss of ambulation.

**Figure 3:**
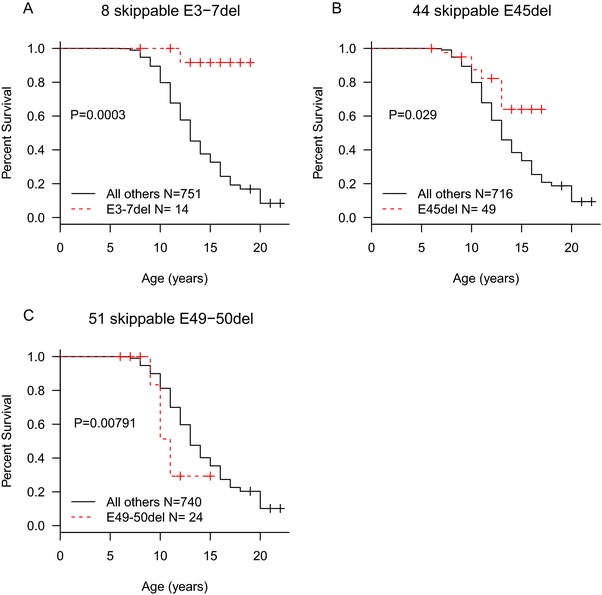
Kaplan–Meier plots for subgroups of exon 8, 44, and exon 51 skippable mutations. (A) Exon 8 skippable patients with exon 3–7 deletions ambulated substantially longer than any other group (*P* = 0.0003). (B) Individuals with single exon 45 deletions ambulate longer than other exon 44 skippable subgroups or other targeted exons (*P* = 0.029). (C) Among exon 51 skippable subgroups, only exon 49–50 deletions show significant change in age at LOA (*P* = 0.008)

Among mutations amenable to exon 44 skip therapy, 64% were due to a single mutation type: exon 45 deletion. Age at LOA in individuals with single exon 45 deletions (*N* = 49) was delayed compared to the other exon 44 skippable mutations and the remainder of the Duchenne Registry population (*P* = 0.029; Figure 3). Thus, these data indicate that the prior observations of exon 44 skippable patients having a milder phenotype may be restricted to the exon 45 deletion subgroup. Exon 3–7 deletions, amenable to exon 8 skipping therapy, were also able to ambulate significantly longer than other groups (*P* = 0.0003). For reasons that are not entirely clear, we also observed that the subgroup of exon 49–50 deletion DMD subjects (*N* = 24) that are amenable to targeted exon 51 skipping therapy were more likely to lose ambulation earlier than all other mutation groups (*P* = 0.008; Figure 3) with a median age at LOA of 11.

We applied Cox regression analysis on 961 individuals to estimate the HR of corticosteroid usage and mutation subgroup on the age at LOA. Exon 44 skippable mutations and exon 8 skippable mutations (e8 skip) were statistically significant explanatory variables for age at LOA (Table 2). Both variables had HR < 1, indicating an effect of delaying age at LOA. Moreover, exon 45 deletions were independently significant predictors of late age at LOA (*P* = 0.015, HR 0.49, CI 0.28–0.87) as well as exon 3–7 deletions (*P* = 0.00125, HR 0.18, CI 0.06–0.51) when included into the previous model. As expected, corticosteroid status was significant for both prednisone (*P* = 0.0077, HR 0.62, CI 0.44–0.88) and deflazacort (*P* < 0.0001, HR 0.30, CI 0.21–0.43), and of a similar effect size as exon 44 skippable mutation status. No other variables were significant.

**Table 2:** Cox regression results using age at LOA as outcome and corticosteroid status and mutation subgroup as covariates

	HR (CI low, high)	*P*
**Current steroids (prednisone)**	0.62 (0.44, 0.88)	**<0.01**
**Current steroids (deflazacort)**	0.31 (0.22, 0.43)	**<0.01**
Discontinued steroids	1.35 (0.94, 1.94)	0.10
**Exon 8 skippable**	0.21 (0.08, 0.53)	**<0.01**
**Exon 44 skippable**	0.54 (0.33, 0.87)	**0.01**
Exon 45 skippable	1 (0.65, 1.55)	0.99
Exon 50 skippable	0.8 (0.46, 1.36)	0.40
Exon 51 skippable	0.99 (0.67, 1.47)	0.96
Exon 52 skippable	1.02 (0.6, 1.72)	0.95
Exon 53 skippable	0.9 (0.59, 1.37)	0.62
Exon 55 skippable	0.92 (0.5, 1.68)	0.78
Duplication	0.99 (0.65, 1.49)	0.95

All variables were entered into a Cox regression model. Significant covariates with *P* < 0.05 are bolded. HR < 1 delays age at LOA. CI, confidence interval.

**Figure 4:**
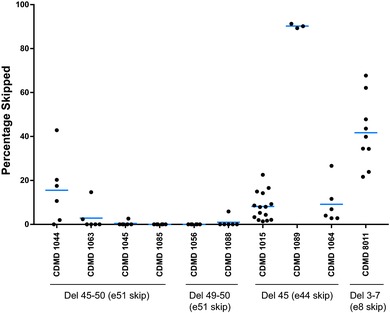
Basal levels of exon skipping are enriched in cultured myotubes derived from reprogrammed fibroblast (iDRM) from patients with del 45 mutations (exon 44 skippable) or myoblasts derived from del 3–7 (exon 8 skippable) relative to those derived from del 45–50 or del 49–50 iDRM (exon51 skippable). Experimental samples were run in triplicate and data shown reflect cumulative results of multiple experiments, with each point representing a singlet.  *DMD* mRNA was reverse transcribed and PCR used to detect exon 44, 51, or 8 skipped and unskipped products. Products were quantitated using a Bioanalyzer. Percentage skipped is calculated as (skipped/unskipped + skipped) × 100

Under the hypothesis that some DMD mutations may result in endogenous skipping to produce an in‐frame mRNA, we quantitated exon skipping in myotube cultures derived from patient iDRM or myoblasts bearing mutations amenable to reframing by skipping exon 51, 45, 44, or 8 available through the CDMD tissue and cell repository. Of note, the representation of DMD mutations in the panel of myotubes examined are largely reflective of the frequencies found in the DMD population: patient mutations reframed by exon 51 skipping comprise the largest cohort, followed by those reframed by exon 45 skipping, exon 44 skipping, or exon 8 skipping, in order of decreasing frequency. We performed reverse transcription PCR on *DMD* RNA extracted from the myotubes and resulting amplicons were quantitated by capillary electrophoresis on an Agilent Bioanalyzer. Five of six iDRM cultures amenable to skipping of *DMD* exon 51 (Figure 4, e51 skip) showed less than 3% of exon 51 skipping, whereas one iDRM demonstrated a high frequency of skipping exon 51 (15%). iDRM lines harboring exon 45 deletions showed exon 44 skipping ranging from 8% to 90%. The myotube culture with an exon 8 skippable mutation also showed high endogenous exon skipping (43%).

**Figure 5:**
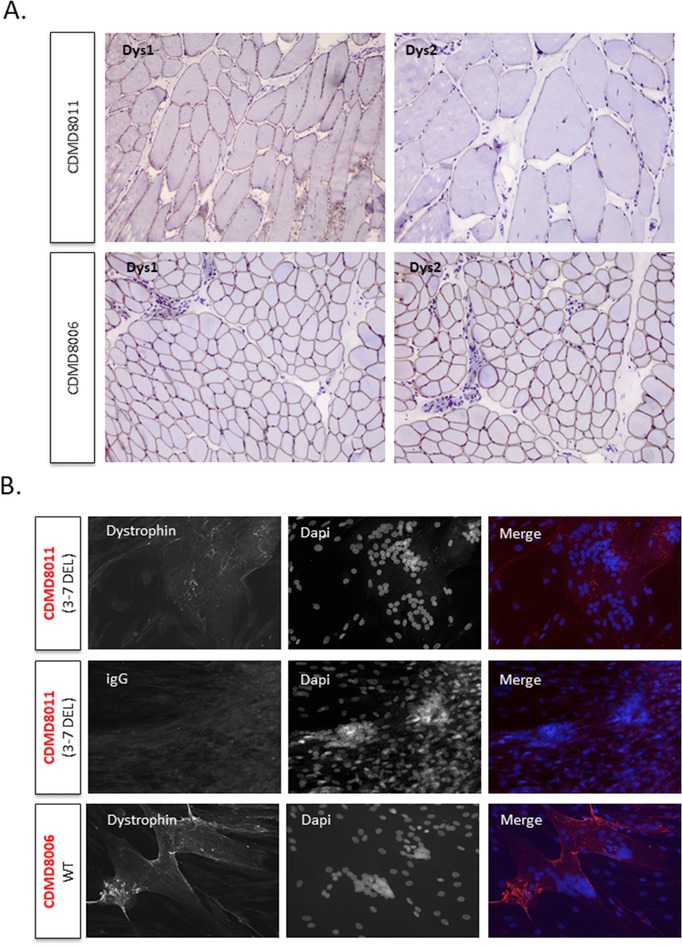
Patient CDMD8011 (del 3–7) expresses low levels of dystrophin protein in muscle biopsy and primary myoblasts expanded and fused to myotubes in culture. (A) Dystrophin is visible at the sarcolemma in transverse sections of muscle in CDMD8011 and CDMD8006 (wild‐type) when stained with DysI (central rod domain) or Dys2 (C‐terminal). Magnification, 10×, 20× for Dys2 CDMD8011. (B**)** Representative images of fused myotubes showed low amounts of dystrophin in CDMD8011 and higher levels in CDMD8006. Mandys8 stains the central rod domain of DMD. Nuclei are colored blue (Dapi). Scale bar 50 μm

In the one instance, where muscle biopsy was performed (CDMD8011), we assessed dystrophin protein levels in both frozen sections and expanded myoblasts fused to myotubes in culture. While deletion of exons 3–7 is predicted to result in a frameshift and loss of dystrophin protein, in keeping with high levels of endogenous skipping observed in the cultured patient myotubes (Figure 4), we found rescued dystrophin protein expression in both transverse sections of frozen muscle biopsy and cultured myotubes (Figure 5).

Our results are consistent with a model in which endogenous exon skipping in *DMD* transcripts results in a low level of in‐frame mRNA and production of low levels of rescued dystrophin protein, which contributes toward reduction in disease severity as measured by delay in age of LOA in exon 44 or 8 skippable patients.

## 4 DISCUSSION

We previously reported the utility of the Duchenne Registry patient registry to observe effects on age at LOA due to single and combination therapies for those affected by DMD (Wang et al., 2014). Furthermore, we demonstrated Duchenne Registry participants were typical of the general DMD population in terms of mutation status, mutation type, or age at diagnosis. Thus, this valuable dataset is highly applicable for observing differences in mutation type within *DMD* based on age at LOA and serving as a reference population demonstrating typical disease course.

The Duchenne Registry records current medication, mutation type, and age at LOA for a large number of participants, allowing us to observe differences in disease severity based on a limited group of mutations potentially amenable to exon skipping. As a web‐based platform, the Duchenne Registry has a relatively low barrier of entry for participants, allowing for a large number of participants. In the available data from the Duchenne Registry, we were able to retain a substantial number (*N* = 765) of males with an exonic duplication, nonsense mutation, or deletion mutation correctable by skipping exon 8, 44, 45, 50, 51, 52, 53, or 55 alongside their steroid usage and age at LOA. The availability of these multiple data types and the ability of participants to update data make the Duchenne Registry a robust data set for exploratory studies and natural history comparison because it is larger than all reported studies investigating DMD age at LOA combined (Bello et al., 2016; Pane et al., 2014; Servais et al., 2015; van den Bergen, et al., 2014).

Our analysis of age at LOA supports the prior observations that not all mutations in the most commonly mutated region of *DMD* are equivalent, and that those amenable to exon 44 (Bello et al., 2016; Koeks et al., 2017; Pane et al., 2014; van den Bergen, et al., 2014) and exon 8 (Bello et al., 2016) skip therapy have a relatively mild disease phenotype, which impacts how clinical trials assessing therapeutic efficacy would need to be designed. In a study from multiple Italian centers, the subgroup eligible for exon 44 skipping had overall longer 6‐minute walk distance (6MWD) than the other deletion mutations, but this result was not significant within a test of heterogeneity (Pane et al., 2014). The lack of statistical significance may be due to the relatively small number of individuals analyzed (*N* = 18 with exon 44 skippable mutations). Two studies from the United States and Netherlands studied age at LOA stratified by AON amenable treatment groups (Bello et al., 2016; van den Bergen et al., 2014). Consistent with the results presented here, both strongly support a delay in age at LOA among individuals amenable to targeted skipping of exon 44.

We estimated the effect of possessing a *DMD* mutation that is amenable to exon 44 skipping to have a hazard ratio (HR) of 0.54. This indicates that the impact of these mutations on the phenotype is roughly comparable with corticosteroid treatment. HRs for deflazacort and prednisone were 0.31 and 0.62, which is consistent with our previous finding that deflazacort tends to have a stronger effect at delaying age at LOA than prednisone (Wang et al., 2014). This is similar to Bello et al. (2016), who estimated HR for exon 44 skippable mutations to be 0.34. Their data also showed deflazacort to be more beneficial in delaying age at LOA compared to prednisone (0.34 vs. 0.22) and that the genetic effect was similar to corticosteroid treatment. These consistencies support the overall utility of the patient self‐report registry model.

In the Duchenne Registry and other registries, exon 45 deletion mutations comprise 65% of DMD subjects who are potentially amenable to exon 44 skipping to restore *DMD* reading frame and represent 4% of all mutations that cause DMD (Aartsma‐Rus et al., 2006; Tuffery‐Giraud et al., 2009). We found exon 45 deletions were the primary driver of delayed age at LOA. When considering exon 45 deletions separately from other exon 44 skippable mutations, the HR in our Cox model decreased from 0.54 to 0.50 and only exon 45 deletions were significant (*P* = 0.02). Several studies have highlighted phenotypic heterogeneity of individuals with mutations around the exon 45–50 hot spot (Deburgrave et al., 2007; Kesari et al., 2008). One biological explanation is that more mildly affected patients may have a higher frequency of revertant fibers due to spontaneous skipping of an exon that generates in‐frame transcripts and resulting functional dystrophin (Dwianingsih et al., 2014; Prior et al., 1997). Although an increased rate of revertant fibers has been demonstrated in some cases (Lourbakos et al., 2011), the true incidence is not known. We found evidence of endogenous exon skipping in patient‐derived iDRMs and myotube cultures amenable to skipping of exons 8 and 44, and this provides a possible biological explanation for the effects observed in the Kaplan–Meier analysis. Baseline and repeat muscle biopsy studies in these patient subgroups with careful dystrophin quantification will shed more light on this as individuals are tested within the context of therapeutic trials intended to restore dystrophin to determine the phenotypic benefit of a low levels of dystrophin in humans. This could serve as a valuable model to determine the phenotypic benefit of low levels of dystrophin in humans. Additionally, iDRM‐derived from DMD patients amenable to reframing by skipping exon 44, 51, or 8 provide culture models for elucidating the molecular basis of enhanced natural skipping and rescued dystrophin production. These would serve as useful adjuncts to ongoing concerns regarding dystrophin quantification.

Among the individuals potentially amenable to exon 8 skipping strategy, deletion of exons 3–7 was the most common mutation reported. HR was 0.21 in the larger group (*N* = 18) and 0.18 in those with exon 3–7 deletions (*N* = 14) and remained statistically significant (*P* = 0.001). Bello et al. estimated exon 3–7 deletions HR to be 0.24, demonstrating remarkable consistency in these sample sets. In both studies, the number of individuals with exon 8 skippable mutations is small, which may limit accurate measurement of the magnitude of effect. We have derived a cell line with deletion of exons 3–7 of *DMD* in which around 10% of *DMD* mRNA demonstrates endogenous skipping of exon 8, which is a potential molecular mechanism for why this group of patients is more mild.

Evidence of a more severe than typical disease course in the exon 51 amenable deletion group has not been reported elsewhere, and the molecular mechanism is not clear at this point. Kaplan–Meier analysis of individual subgroups within exon 51 amenable mutations revealed that exon 49–50 deletions appeared to be particularly severe with a median age at LOA of 11 years versus 13 years for the rest of the cohort (*P* = 0.008). However, this result was not significant in the Cox analysis.

Based on these findings, the exon 8, 44, and 51 skippable subgroups may serve as inappropriate natural history controls for most exon‐skipping trials, but that other mutations amenable to exon 45, 50, 52, 53, 55, and some 51 or nonsense mutations are generally comparable and can reasonably serve as contemporary natural history controls. Even in instances where restoration of dystrophin is not the intended effect, the underlying genetic mutation may need to be considered to prevent the confounding of genetic effects within the potential therapeutic effect being sought. Thus, more natural history data may be necessary to improve the sensitivity of ongoing clinical trials for DMD in order to appropriately factor *DMD* mutation effects into clinical trial designs, and exact mutation type in the *DMD* gene should be considered in data interpretation. It is possible that restriction in clinical trial entry based on mutation type could reduce subject variability and enhance the ability to observe a study drug effect.

Because much of the Duchenne Registry data are based on patient and parent self‐reports, there is sometimes concern regarding accuracy relative to natural history studies derived from academic medical centers where participants are recruited, phenotyped and followed longitudinally by expert clinicians. Although care has been taken to rule out inconsistencies and errors in responses (for instance, DMD genetic reports are reviewed and inputted by certified genetic counselors), misunderstandings and errors can occur in the survey questionnaire. We also note that the retrospective nature of this study and its internet‐based recruitment methods may also influence the demographics of respondents, which may tend toward more technically literate families. However, the remarkable consistency of the findings reported here and the recently reported support for the observed superiority of deflazacort relative to prednisone (Griggs et al., 2016; Wang et al., 2014), serve to further validate and increase confidence in this important resource.

Using a large patient self‐report registry, we have linked subgroups of genetic mutations to a critical and irreversible physiological milestone in DMD. These results indicate further study of exon 3–7 and exon 45 deletion subjects, which both show delayed age at LOA, will likely produce further understanding of the structure and function of aberrantly affected *DMD* transcripts and naturally occurring mitigating factors. Furthermore, the lack of equivalence in disease progression between different mutation subgroups that is apparent with the large sample set suggests caution when used for clinical trials or as natural history or external contemporary controls. However, the consistency of the age at LOA across multiple mutation types provides a powerful way to determine if long‐term administration of new therapeutics is causing deviation from expected disease course. This will become increasingly important as drugs such as Exondys51 gain approval through the accelerated approval pathway based on a reasonably likely to predict clinical benefit standard.

## CONFLICT OF INTEREST

The authors have no conflicts of interest to report.

## Supporting information


**Figure S1**. Kaplan–Meier LOA analysis for patients eligible for skipping therapy of exons (A) 8, (B) 44, (C) 45, (D) 50, (E) 51, (F) 52, (G) 53, (H) 55, (I) exonic duplication and (J) nonsense mutations. Delayed LOA was among individuals amenable to exon 8 skipping (*P* < 0.001) and exon 44 skipping (*P* = 0.03). Exon 51 skippable individuals had earlier LOA (*P* = 0.04). All subjects were currently using corticosteroids.
