# Supplementary material for: DMD genotype correlations from the Duchenne Registry: Endogenous exon skipping is a factor in prolonged ambulation for individuals with a defined mutation subtype
Source: Hum Mutat. 2018 Jul 12;39(9):1193–202. doi: 10.1002/humu.23561 (PMC6175390; doi:10.1002/humu.23561)
Supplement: Supplementary file 1 — Figure S1. Kaplan–Meier LOA analysis for patients eligible for skipping therapy of exons (A) 8, (B) 44, (C) 45, (D) 50, (E) 51, (F) 52, (G) 53, (H) 55, (I) exonic duplication and (J) nonsense mutations. Delayed LOA was among individuals amenable to exon 8 skipping (P < 0.001) and exon 44 skipping (P = 0.03). Exon 51 skippable individuals had earlier LOA (P = 0.04). All subjects were currently using corticosteroids. [file HUMU-39-1193-s001.docx]

**
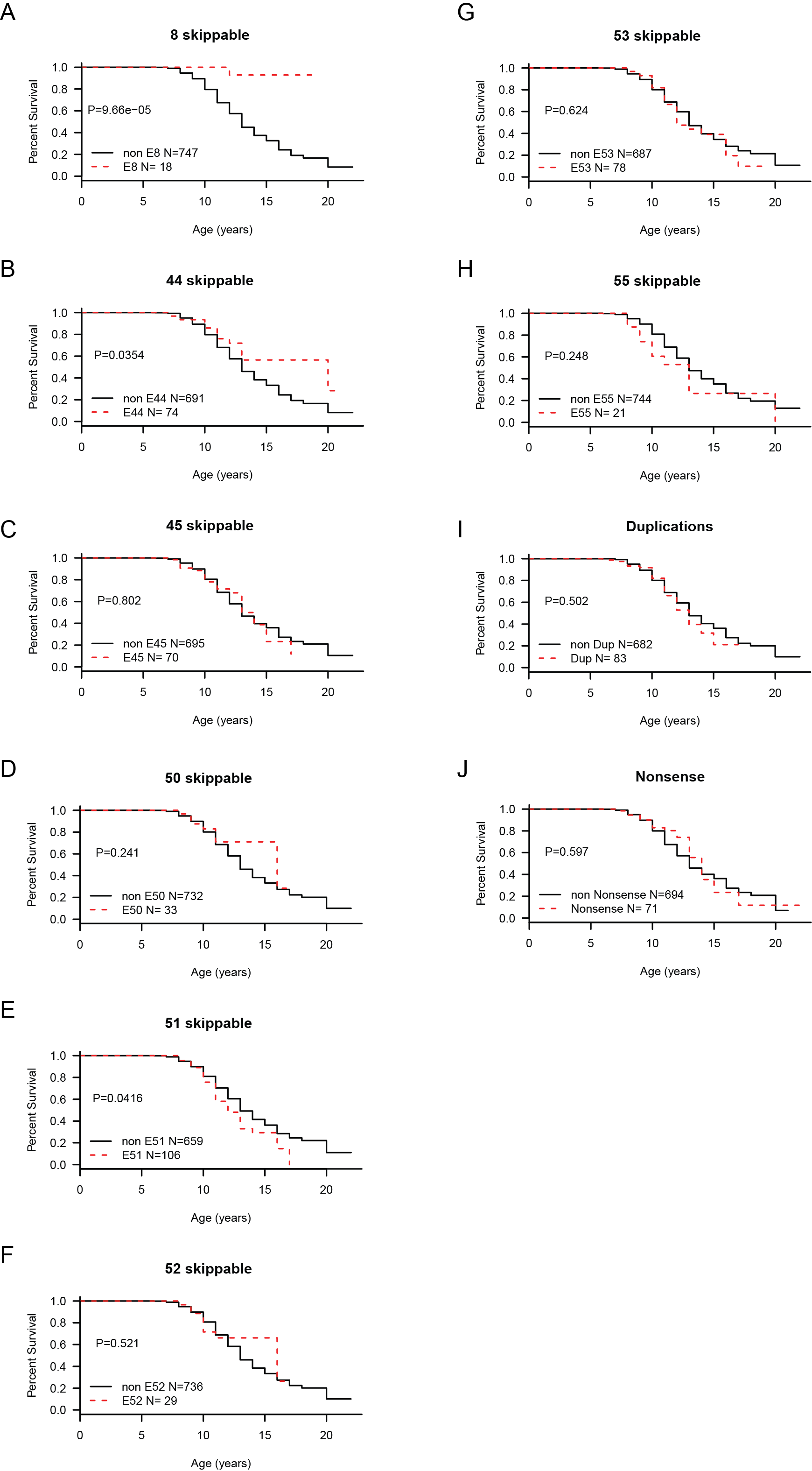
**

**Supp. Figure S1.** Kaplan Meier LOA analysis for patients eligible for skipping therapy of exons (A) 8, (B) 44, (C) 45, (D) 50, (E) 51, (F) 52, (G) 53, (H) 55, (I) exonic duplication and (J) nonsense mutations. Delayed LOA was among individuals amenable to exon 8 skipping (P<0.001) and exon 44 skipping (P=0.03). Exon 51 skippable individuals had earlier LOA (P=0.04). All subjects were currently using corticosteroids.
